# An Apple a Day Keeps the Doctor Away: Potential Role of miRNA 146 on Macrophages Treated with Exosomes Derived from Apples

**DOI:** 10.3390/biomedicines10020415

**Published:** 2022-02-10

**Authors:** Martina Trentini, Federica Zanotti, Elena Tiengo, Francesca Camponogara, Margherita Degasperi, Danilo Licastro, Luca Lovatti, Barbara Zavan

**Affiliations:** 1 Department Translational Medicine, University of Ferrara, 44121 Ferrara, Italy; martina.trentini@unife.it (M.T.); federica.zanotti@unife.it (F.Z.); elena.tiengo@student.unife.it (E.T.); francesca.camponogara@unife.it (F.C.); 2 CIF Consorzio Innovazione Frutta, Via Edmondo Mach 1, 38010 San Michele all’Adige, Italy; lovatti@cif.tn.it; 3 AREA Science Park, Padriciano, 99, 34149 Trieste, Italy; margherita.degasperi@areasciencepark.it (M.D.); danilo.licastro@areasciencepark.it (D.L.)

**Keywords:** apple, miRNA, macrophages, exosomes

## Abstract

The constant dialogue between the plant world and the animal world (including man among them) has been known since the time of Adam and Eve, where an apple was the origin of the evils of the world. Apart from Snow White—who might have something to object to when it comes to the use of apples—fruits, plants, and natural extracts have been known for millennia as remedies for human health-related ailments. In the light of such evidence, the aim of the present work was to investigate from a biological point of view the potential role of apple exosomes in inflammatory processes on human cells. To this end we isolated and characterized apple exosomes and treated human cells such as macrophages and NCTC L929 as cancer cells in order to evaluate the tumorigenic and anti-inflammatory effect of apple exomes. Microscopic and molecular biology analyses were conducted to characterize exosomes and to assess cell proliferation, death, and miRNA line, as well as gene expression and the uptake of exosomes by cells. The results confirm the absolute biological safety of exosomes and their anti-inflammatory effect, mediated mainly by miRNA146 production by M2 macrophages.

## 1. Introduction 

Cells are able to communicate with each other through numerous techniques, such as cell–cell contacts, cell–matrix contacts, receptors, open junctions, extracellular signals, etc. Recently the attention of researchers has been focused on a new type of communication: through extracellular vesicles. These vesicles, nano-sized, are a kind of email that all cells use to communicate with each other at a distance. They are in fact lipid bilayer vesicles with protein receptors on the outside that determine the sender (cells from the source), the recipient (cell designated to receive them), and high information content, mainly consisting of miRNA instructions to change the biological behavior of the receiving cell [1,2,3,4,5]. 

Vegetables are also able to produce these extracellular vesicles with highly informative content: the plant-derived nanovesicles (PDNVs). These are nano-sized membraned vesicles ranging from 30 nm to 1000 nm that have been isolated, physically and biologically characterized in plant tissue of several species, including for instance lemon [6], strawberry [7], sunflower seeds [8], A. thaliana [9], and apple [10].

In addition to lipidic composition, recently PDNVs RNA content has received great attention in order to define their biological effect [11,12,13]. PDNVs can carry small RNAs, messenger RNAs, cytosolic proteins, and other small organic molecules such as vitamin precursors. Although to our knowledge they are primarily used as defense mechanisms towards fungal infection in plants [14,15], their capability to be transported in the phloem and to bypass biological barriers indicates a possible role in RNA transport and plant cell communication [16]. However, edible PDNVs have also been observed in inter-species interactions. Several studies have provided evidence of PDNVs’ cross-kingdom regulation in mammalian models [17]. Firstly, a plant-derived miRNA (miR-168a) was found in rice (*Oryza Sativa*)-fed mice’s circulation, enclosed in lipidic vesicles, suggesting that PDNVs can survive the extreme environment in the digestive tract [18], which was further confirmed to be true for other PDNVs [19]. The long persistence in biological fluids and lack of generated cytotoxicity in mammalian models suggest that PDNVs are highly biocompatible with life [20,21]. It is well-known indeed that the dietary health benefits of fresh fruits and vegetables have been reported in countless studies, through the careful observation of each component’s relationship with the digestive tract [22,23,24,25]. These results suggest that PDNVs have similar biochemical characteristics to mammalian exosomes and then that they could target mammalian tissue (such as intestinal epithelial cells, hepatocytes, and Caco.2 intestinal cells). Edible PDNVs exhibit intrinsic antioxidant and anti-inflammatory activity in recipient mammalian cells due to their specific content [26,27]. Particularly, a Zhang et al. study on ginger observed PDNVs’ ability to mediate important physiological functions in the mammalian digestive tract, reducing inflammatory bowel disease (IBD) and colon tumor growth [19] thanks to their miRNA cargo.

In light of such considerations, the aim of the present study was to test whether extracellular vesicles derived from apples are able to activate a direct and active dialogue with mammalian cells. In this view, we used apple-derived nanovesicles (ADNVs) isolated from Malus domestica, Golden Delicious variety. ADNVs were characterized in terms of morphological features (size, distribution, morphology) and tested in three different cell types, such as a cancer cell line, fibroblasts, and macrophages, in order to verify their safety and their biological role.

## 2. Materials and Methods

### 2.1. ADNVs Isolation and Purification

Starting material comprised *Malus Domestica* sp. Variety Golden Delicious, cultivated in Val Di Non (TN), Italy. Two apples of approximately 250 g each were washed thoroughly and smashed into a pulp. The pulp was then homogenized and subjected to a series of centrifugations at increasing speed (650× *g* 5 min, 3000× *g* 10 min and 10,000× *g* 10 min), after which the pellet was discharged. The supernatant fraction was then filtered with 0.2 um syringe filters (GVS S.p.A, Bologna, Italy) and centrifugated at 15,000× *g* with an Ultracentrifuge Optima L-70 (Beckman Coulter Inc., Brea, CA, USA), type 70 Ti rotor, to remove smaller particles and debris. The supernatant was further centrifugated at 110,000× *g*, the resulting pellet was resuspended in 1 mL PBS (Thermo Fisher Scientific, Waltham, MA, USA), and was used as ADNVs fraction in all following experiments. The ADNVs fraction was conserved at –80 °C until use.

### 2.2. Size Distribution and Concentration Measurement of ADNVs 

The concentration and hydrodynamic radius distribution of ADNVs was analyzed with tunable resistive pulse sensing technique, or TRPS (qNANO, Izon Science Ltd., Cambridge, MA, USA). This required a NP100 nanopore at a stretch of 47 mm. Particle’s concentration was standardized using multi-pressure calibration (10 amp and 20 amp) with CPC100 at a concentration of 1.7 × 10^13^ particles/mL and average hydrodynamic radius of 100 nm (Izon Science Ltd., Cambridge, MA, USA). All measurements were carried out in triplicates.

### 2.3. Cell Culture

The THP-1 monocyte cell line was bought from Resnova (Rome, Italy) and was cultured in RPMI 1640 medium [-] L Glutamine (Thermo Fisher Scientific, Waltham, MA, USA) with the addition of 2 mM L-Glutamine (Euroclone S.p.a, Pero, Italy) and 10% FBS (Euroclone S.p.a, Pero, Italy). Cell cultures were maintained at 37 °C and 5% CO_2_, and the medium was changed twice a week. For all experimental purposes, differentiation of THP-1 monocytes into macrophages was carried out with complete RPMI 1640 medium containing 100 ng/mL of Phorbol 12-myristate 13-acetate (PMA) for a period of 24 h. Subsequently, a resting period of 72 h was allowed before treatments, in which adhered macrophages were cultured with complete RPMI 1640 medium [28]. Fibroblasts and NCTC L929 cell lines were also purchased from Resnova (Rome, Italy) and were cultured with DMEM medium (Euroclone S.p.a, Pero, Italy) with 10% FBS and 1% antibiotic–antimycotic (Euroclone S.p.a, Pero, Italy). Fibroblasts and macrophages were further treated with TNFα (10 ng/mL) and IFNg (50 ng/mL) for 6 h to induce inflammation.

Treatment with ADNVs fraction was performed by adding an amount of ADNVs solution mounting to a concentration of 8 × 10^10^ particles/mL to cultured cells. The control group was instead constituted by untreated cells. 

### 2.4. Transmission Electron Microscopy

For the morphological analysis of isolated ADNVs, a fixating solution of 2% glutaraldehyde in phosphate buffer was added in a 1:1 proportion to the ADNVs fraction. ADNVs were then deposited, rinsed, and stained with heavy metal compounds onto a gridded slide following standard protocols. The slide was then visualized with a TEM Zeiss EM 910 instrument (Zeiss, Oberkochen, Germany).

### 2.5. Scanning Electron Microscopy

A morphological analysis of cultured cells was carried out through 2D SEM imaging and was performed by Centro di Microscopia Elettronica of Ferrara University (Ferrara, Italy) with SEM Zeiss EVO 40 (Zeiss, Oberkochen, Germany) instrument. For this experiment, 3 × 10^5^ THP-1 cells were seeded onto sterile, poly-d-lysine coated coverslips and differentiated into macrophages. After treatment, all samples were fixed with 2% glutaraldehyde in phosphate buffer at 4 °C, dehydrated with baths of increasing ethanol concentrations, mounted, and sputter-coated with gold, following standard protocols. Imaging was performed under high vacuum condition using a secondary electron detector [29].

### 2.6. LDH and MTT Assays

For both assays, THP-1 cells were seeded at a concentration of 5 × 10^5^ cells/well and treated as mentioned above. Both intracellular and extracellular LDH activity was assayed with LDH Activity Assay kit (Sigma-Aldrich, St. Louis, MO, USA) in treated and control samples after 24 and 72 h of treatment. The culture medium was collected for the analysis of extracellular LHD activity, while intracellular activity was measured after cell lysis with the kit’s LDH Assay buffer. Each sample was centrifugated at 10,000× *g* for 15 min at 4 °C, and 50 µL was used for three replica measurements. Absorbance was measured at 450 nm with a multilabel plate reader (Victor 3, Perkin Elmer, Milano, Italy). The NADH Standards for colorimetric detection and positive control were carried out following manufacturer’s instructions. All conditions were tested in triplicates.

To test cell proliferation, THP-1 and NCTC L929 cell samples were assayed after ADNVs incubation. NCTC cells were seeded at a concentration of 40 K cells/mL. All samples were incubated with 1 mL of 0.5 mg/mL MTT (3-(4,5-dimethythiazol-2-yl)-2,5-diphenyl tetrazolium bromide) solution in PBS for 3 h at 37 °C. The MTT solution was then removed, and the formazan cell content was extracted with 0.5 mL of 10% DMSO. OD values were recorded at 570 nm for each sample in duplicate, for a volume of 200 µL each, using a multilabel plate reader (Victor 3, Perkin Elmer, Milano, Italy). This experiment was performed three times independently.

### 2.7. Fluorescence Labelling and Imaging

After treatment, THP-1-derived macrophages seeded and treated on poly-d-lysine coated coverslip were fixed with 4% PFA solution. Excess of PFA was quenched by washing with glycine 0.1 M in PBS, cells were then permeabilized with Triton-X 100 0.1% in PBS and saturated with a solution of BSA 2% in PBS. Primary antibodies were applied overnight as follows: 4 µg/mL iNOS mouse monoclonal antibody; 5 µg/mL CD68 mouse monoclonal antibody (Thermo Fisher Scientific, Waltham, MA, USA). All primary antibodies were diluted in 2% BSA, 0.1% Triton-X in PBS. Samples were then covered with appropriate secondary antibodies: 4 µg/mL goat anti-rabbit IgG Alexa Fluor 633 (Invitrogen, Thermo Fisher Scientific, Waltham, MA, USA) for iNOS-labelled cells and 2 µg/mL goat anti-mouse IgG Alexa Fluor 647 (Invitrogen, Thermo Fisher Scientific, Waltham, MA, USA) for CD68 labelled cells. Cytoskeleton staining was also obtained, by adding phalloidin Alexa Fluor 488 (Thermo Fisher Scientific, Waltham, MA, USA) to each secondary antibody solution as instructed by the provider. Lastly, cell nuclei were stained with Hoechst fluorescent dye (Sigma-Aldrich, St. Louis, MO, USA) following the provider’s instructions.

ADNVs were stained with PKH26 membrane labelling fluorophore (Sigma-Aldrich, St. Louis, MO, USA). The protocol furnished by the manufacturer was optimized for nanoparticles staining as follows. Briefly, 0.8 µL PKH26 in 200 µL Diluent C was added to PBS or to the ADNVs fraction, resuspended in Diluent C after isolation, for a final volume of 400 µL. After resuspension, the mix was incubated for 5 min at RT to allow staining; the mix was subsequentially moved to 30 K membrane centrifugal filters (Amicon Ultra-0.5, Millipore, Burlington, MA, USA) and centrifugated at 14,000× *g* for 20 min to discharge excessive dye. Stained ADNVs and PBS were used for cell line treatment as described above. At the end of the experimental treatment, cells were fixed with 4% PFA and stained with phalloidin Alexa Fluor 488 (Thermo Fisher Scientific, Waltham, MA, USA) according to manufacturer’s instructions. Imaging was performed with confocal microscope Nikon ECLIPSE Ti, DS-Qi2 camera, 40 x air and 60 x immersion objectives. 

### 2.8. RNA Extraction, Sequencing, and RT-qPCR

Total RNA extraction was performed on THP-1-derived macrophages and fibroblasts (w/wo TNFα and IFNg) after treatment using Total RNA Purification Plus Kit (Norgen Biotek Corp., Thorold, ON, Canada) following manufacturer instructions for cells growing in monolayer. The extracted RNA quality and concentration was verified with NanoDrop One (Thermo Fisher Scientific, Waltham, MA, USA). RNA was then stored at −80 °C until use.

Sequencing of all miRNAs was carried out by Area Science Park (ASP, Trieste, Italy) with Illumina sequencing.

MiRNA-Seq libraries were prepared using the QIAseq miRNA Library Kit (QIAGEN; Hilden, GE) and sequenced using Novaseq 6000 (Illumina; San Diego, CA, USA) in 2 × 150 paired-end mode. Identification of miRNAs in the samples was performed using the QIAseq miRNA-NGS data analysis software considering Single Read as read type and Read 1 Cycles 75 as read cycles.

To determine differential genetic expression, first strand cDNA was synthetized from total RNA. For each sample, 1200 ng of RNA was reverse transcribed with SensiFAST cDNA Synthesis Kit (Meridian Bioscience, Cincinnati, OH, USA) in a final volume of 20 µL. Real-time quantitative PCR was performed with primers reported in Table 1, with GDS Rotor-Gene^®^ Q Thermocycler (QIAGEN, Hilden, Germany) using SensiFAST SYBR No-ROX master mix (Meridian Bioscience, Cincinnati, OH, USA) according to manufacturer instructions. Thermal cycling and fluorescence detection were carried out as follows: PCR activation step at 95 °C for 2 min, followed by 40 repeating cycles of denaturation (95 °C for 5 s), annealing (60 °C for 10 s), and extension (70 °C for 20 s). Lastly, melting temperature of amplicons was analyzed with a further step of growing temperature from 72 °C to 95 °C in a span of 5 min. Each experiment was repeated in triplicates, and each measurement was taken three times.

### 2.9. Statistical Analysis

All results are expressed as a mean, with an indication of the standard error (SE) obtained from at least three independent replicas of the experiment. Significant difference between groups was determined by analysis of variance (ANOVA) and multiple comparisons by post hoc Bonferroni test. Statistical significance is labeled as follows: * *p* < 0.05, ** *p* < 0.01, *** *p* < 0.001, and **** *p* < 0.0001.

## 3. Results

### 3.1. ADNV Characterization

**Figure 1:**
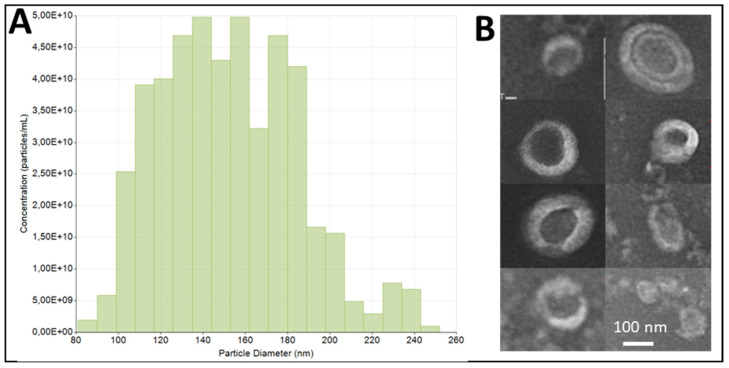
ADNVs size distribution. The graph (**A**) represents the size distribution and quantification of the APNP fraction, analyzed by tunable resistive pulse sensing (qNano). The average hydrodynamic diameter is 152 nm (St. Dev ± 32.3 nm), while the average concentration of three measurements is 4.78 × 10^11^. TEM images show ADNVs morphology and size (**B**).

ADNPs isolated were analyzed in order to define size and distribution by means of size-distribution analysis (Figure 1A) and TEM analyses (Figure 1B). Results confirm that isolated ADNPs have an average radius of 152 ± 32.3 nm (Figure 1A) in a range from 90 to 180 nm. These results were also confirmed by TEM (Figure 1B), in which their features were morphologically homogeneous, round-shaped, with a size variability ranging from 80 to 250 nm). 

### 3.2. Uptake of ADNVs

**Figure 2:**
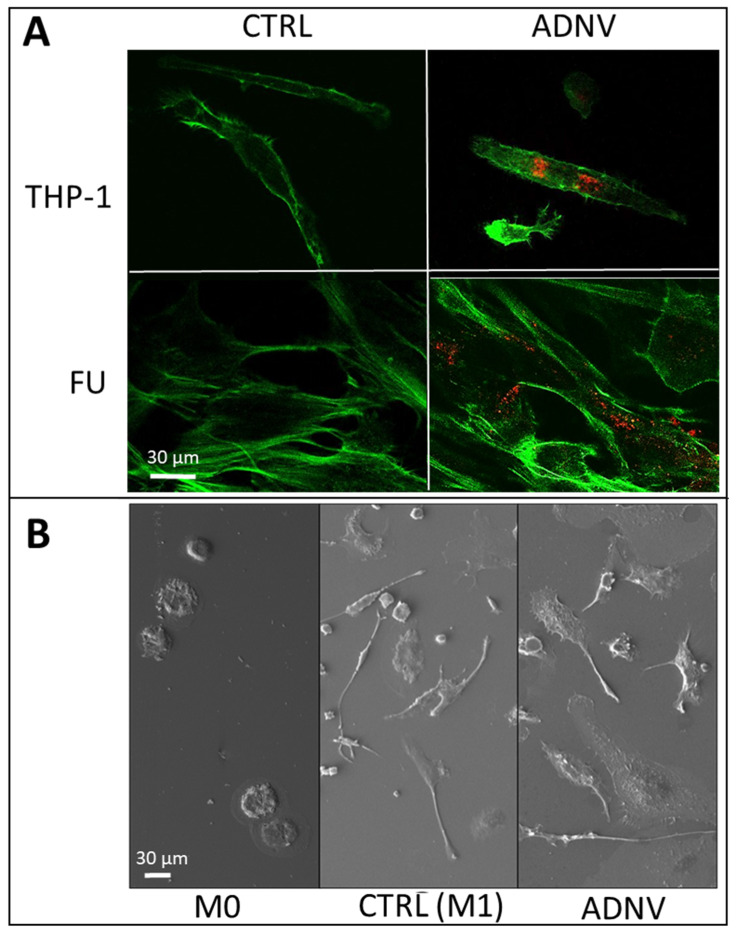

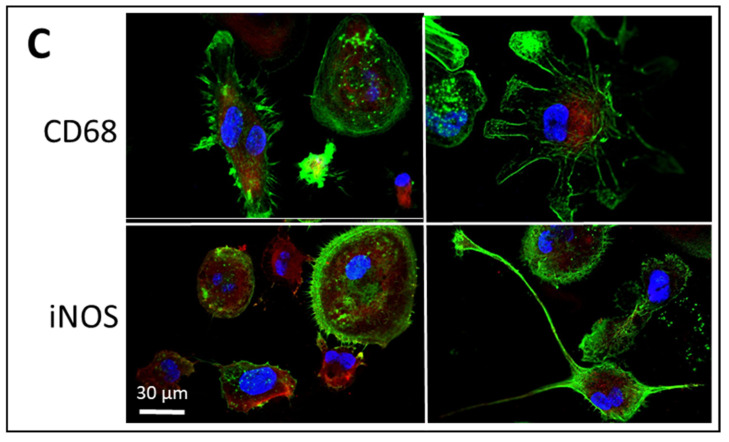
(**A**) ADNVs stained with fluorescent dye (in red) have been absorbed by THP-1 derived macrophages and fibroblasts (FU) stained in green. (**B**) SEM microscopy images show the morphology of non-active (M0), control, and ADNVs-treated macrophages. (**C**) THP-1 macrophages’ cytoskeleton is stained in green and nuclei in blue; red fluorescence indicates iNOS and CD68 immunostaining, respectively.

ADNVs were stained with PKH26 dye for a qualitative analysis of their interaction with living cells using fluorescence microscopy. As shown in Figure 2, ADNVs stained in red are present as being spot-like in the cytoplasm of THP1 and on fibroblasts (FU) used as recipient cells. To identify the polarization state of THP-1-derived macrophages, we performed an immunofluorescence evaluation of MΦ and M1 macrophages phenotypical markers, CD68 and iNOS, respectively. The presence of inflammatory factor induced an activation of THP1 on macrophages type I, as confirmed by SEM analyses (Figure 2B) that show the morphological changes of the cells. Macrophages in the two conditions seem to have different shapes, the control being more fuse-like while the ADNVs-treated sample has a more spread-out cytoplasm. Spindle-like morphology has been associated with activated M1 macrophages, while a more round and broad shape is an indication of alternatively activated M2 phenotype [30].

Moreover, this communication was evaluated by immunofluorescence staining against macrophages type 1 markers (CD68 and iNOS). The presence of red spots inside the cytoplasm confirmed that macrophages type I were also able to uptake the ADNVs (Figure 2C).

### 3.3. ADNVs Effects on Cell Viability

**Figure 3:**
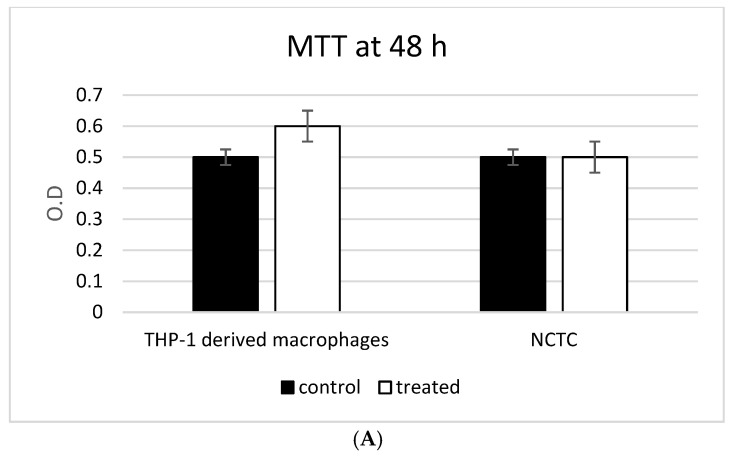

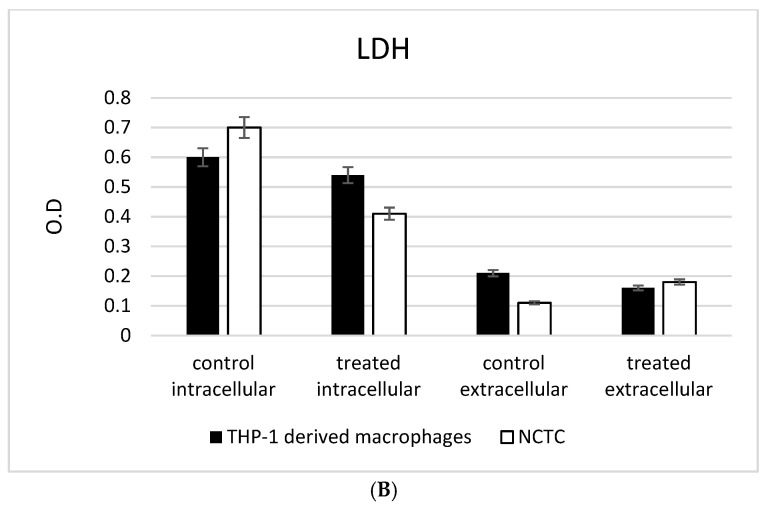
(**A**) Cell proliferation results of MTT assay. In the graph are reported absorbance values for MTT assay on THP-1 and on NCTC L929 after 24 h incubation with and without ADNVs (**B**) Graphic representation of LDH activity inside and outside the cell, after 24 h of incubation with and without the ADNVs. No statistically relevant difference was found.

In order to evaluate their ability to affect cell viability, THP-1-derived macrophages and NCTC L929 cells were incubated with isolated ADNVs. An MTT test to evaluate mitochondrial physiology was performed (Figure 3A). The results from MTT test show a significantly higher proportion of viable cells in ADNVs-treated THP-1 samples compared with the control after 24 h exposure (Figure 3A). The NCTC L929 cell absorbance value of MTT test was similar after 24 of exposure. The effect on damage of the plasma membrane were performed by means the measuring of LDH activity inside the cells and outside the cells. In presence of damage, the cells’ release outside the enzyme could be evaluated by the test. As reported in Figure 3B, LDH is well present inside both cell types and quite absent in the extracellular environment, confirming that the ADNVs did not induce any kind of cell damage.

### 3.4. Differential miRNA Expression Analysis and Target Gene Prediction

**Figure 4:**
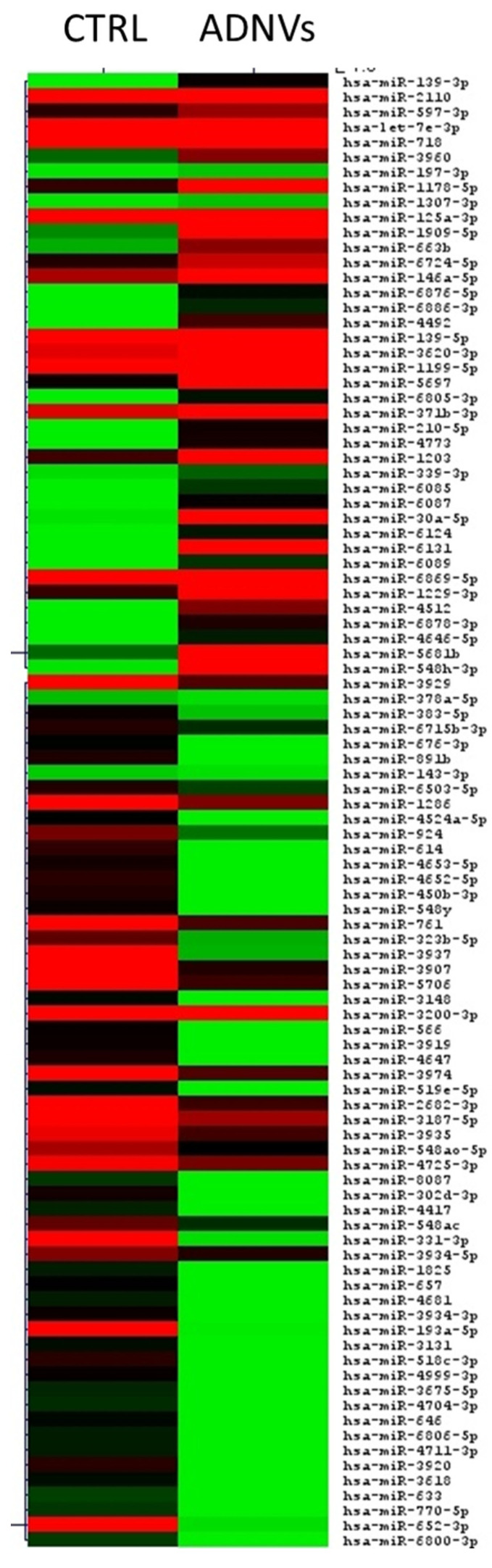
Heatmap of miRNAs expression in control and ADNV conditions.

In total, 2155 miRNAs were identified by next-generation sequencing (NGS) in the 6 samples (Figure 4), of which 10 were significantly upregulated and only three significantly downregulated. 

**Figure 5:**
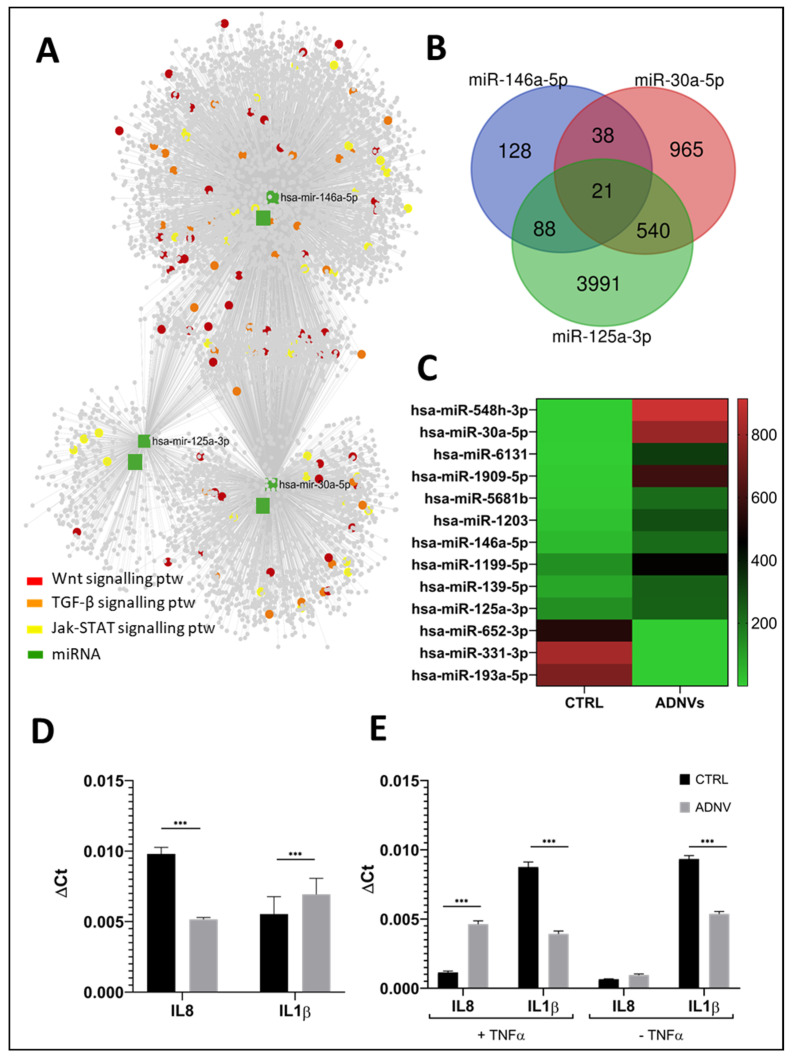
(**A**,**B**) Enrichment analysis of differentially expressed miRNAs with miRNet software. The first graph shows the network of miRNAs and their targeted genes; a few key elements are highlighted in different colors for different metabolic pathways in which they are involved. (**B**) Graph is instead a visualization of genes shared as targets by miR-146a-5p, miR-125a-3p, and miR-30a-5p. (**C**) Heatmap representation of statistically significant, differentially expressed miRNAs in THP-1 control samples and THP-1 incubated with the ADNVs fraction for 48 h, from less expressed (green) to more expressed (red) in both conditions. (**D**,**E**) The two graphs show expression values (ΔCt) results with RT-qPCR of IL-1b and IL-8 cytokines in THP-1 (**D**) and fibroblast with and without previous TNFα treatment (**E**). *** *p* < 0.001.

The differentially expressed miRNAs are shown in Table 2, while the heatmap representation is shown in Figure 5A. Gene targets of differentially expressed miRNAs were compared using miRNet software. The total load of cross-regulated genes for differentially expressed miRNAs is of 10,438. Among miRNAs, the most connected nodes were certainly miR-146a-5p (degree of 4469), followed by miR-30a-5p (degree of 2265) (Figure 5B). After miRNAs enrichment analysis, we found five out of thirteen miRNAs had known functions in cell metabolism, of which three were involved in inflammation, immune response, apoptosis, and cell proliferation/tumor suppression (see Table 2). Among miRNAs reported in Table 2, miR-125a, miR-30a, and miR-146a share the most interesting functions. Cross-linked information between miRNet software, KEGG, and Gene Ontology databases helped identify their targeted genes and roles. In Figure 5A are reported all genes of interest involved in these cellular mechanisms. Possible gene targets, researched with TargetScan Human software, were compared to find that 21 genes were targeted by all three miRNAs (Figure 5B).

Marker genes expression for pro-inflammatory (M1) phenotype polarization was also assayed both in control and 48 h treatment with ADNVs, through RT-qPCR (Table 1). Interleukin 8 (IL-8) and interleukin 1b (IL-1b) were used as M1 markers. Figure 5D clearly shows significant downregulation of both IL-8 and IL-1b in ANDVs-treated samples. Inflammation-related genetic expression was also observed in fibroblasts, both in control and ADNVs-treated conditions (Figure 5E). Gene regulation was assayed for two conditions, with (+TNFα) and without (−TNFα) the addition of TNFα in the growth medium. TNFα induces the expression of IL-1b and IL-8 inflammatory cytokines [31]. In this case too, IL-8 and IL-1b gene expression was assayed to portray indication for general inflammation. The relative expression shows a downregulation of IL-1b in ADNVs-treated samples, both in the presence and absence of inflammation. However, IL-8 levels were significantly higher in +TNFα and ADNVs-treated samples and were comparable in –TNFα samples.

## 4. Discussion

The aim of the present study was to define biological properties of extracellular vesicles isolated from apple—namely, ADNVs. As a first test, we characterized Golden Delicious—derived in size and shape—observing a mean radius of 157 nm and a homogeneous, round shape similar to mammalian exosomes (Figure 1 and Figure 2). The results are in line with PDNVs described from other fruits and vegetables [6,19,20,32]. 

The biological effect of ADNVs has been in the end evaluated focusing our attention on their potential role on general inflammatory process; to this end, we used THP-1 and FU. Their ability to talk with mammalian cells we evaluated through the presence of ADNVs inside the cytoplasm of the cells after incubation with the extracellular vesicles. On both cell lines used (fibroblast and THP-1), red spots related to ADNVs previously stained in red are clearly present in all cytoplasm. Figure 3A shows indeed that red fluorescent ADNVs were up taken by the cells and that they were distributed in the cytoplasm. Our results confirm that mammalian cells are able to recognize ADNVs and, thanks to this positive interaction, they are able to act on their intake process. Moreover, their primary biological activity was evaluated through the mitochondrial activity of the cells with a MTT test and through the evaluation of the plasma membrane damage with a LDH test. These tests were carried out in order to define their cytotoxicity. Both tests, performed also on cancer cell lines, confirmed the safety of the treatment with ADNVs (Figure 3A,B). 

The subsequent evaluation of their biological effect was then a focus on a specific process: the inflammation. To this, we focused our attention on macrophages. Macrophages are involved in the first innate immune response against pathogens and are responsible for tissue inflammation regulation through the production of pro- and/or anti- inflammatory cytokines. Depending on their purpose, inactivated macrophages (M0) can therefore differentiate in two antipodal phenotypes: active macrophages (M1, pro inflammation) and alternative active macrophages (M2, promoting wound healing) [33]. Macrophages often exhibit phenotypes in between the M1 and M2 polarization; nevertheless, macrophages’ cytokine production influences the immune system response against infection [33,34]. 

In this view we created an inflammatory environment in order to drive the activation of THP-1 cells on macrophages type I. This activity was evaluated by means of the gene expression related to inflammatory cytokines from macrophages type I and their complete miRNA evaluation after treatment. Gene expression of inflammatory cytokines reported in Figure 5 show that the treatment of ADNVs on macrophages type I induced a decreased expression of IL-1b and IL-8 (Figure 5). IL-1b is a potent pro-inflammatory cytokine; it stimulates prostaglandin synthesis, neutrophile activation, and other cytokines productions [35,36]. Together with TNFα, they induce the canonical NF-kb (Nuclear Factor kappa-light-chain-enhancer) pathway [37]. NF-kb is a family of transcription factors with roles in several cellular mechanisms such as immune response and development, apoptosis regulation and cell survival, mediating responses from various diverse stimuli [38]. Macrophage’s stimulation of NF-kb cascade leads to their activation, pro-inflammatory cytokines, and chemokines release, among which is IL-8 [37]. Countless studies, both in vivo and in vitro, have underlined the mir-146a role in negatively regulating the production of pro-inflammatory cytokines by inhibition of the NF-kb pathway [39,40,41,42,43,44]. Our results related to miRNoma analyses reveal the higher expression mainly for miRNA 146.

The main action of miR-146a and miR-146b is to inhibit IRAK1 and TRAF6 mRNA transcription in the early stages of inflammation [45,46,47,48]. The overexpression of miR-146a, coupled with downregulation of IL-1b, suggests the repression of the NF-kb pathway in activated macrophages after ADNVs exposure.

In later stages of macrophages activation, miR-125a and let-7e also come into play to suppress NF-kb-induced response and therefore start the healing process [46]. Our data also show that miR-125a is significantly overexpressed, while let-7e approaches a significance threshold (Figure 5C). MiR-125a also suppresses classical M1 activation in macrophages and can suppress bactericidal activity [41,49]. A recent study suggests that miR-125a represses the expression of 5-lipoxygenase, which is involved in the production of leukotrienes and so important for the anti-inflammatory process [49].

Further proof of ADNVs effect on the anti-inflammatory response was tested by monitoring the genetic expression of fibroblasts treated with ADNVs. Fibroblasts have an active role in tissue repair, wound healing, and inflammation and can also be considered immunoregulatory cells [50,51]. ADNVs induce a reduction in inflammatory cytokines in −TNFα samples (Figure 5E), consistent with THP-1-related results. This demonstrates that ADNVs can act from different angles, inducing anti-inflammation signals in multiple cell lines. 

In addition to NF-kb pathway induction, TRAF6 is also associated with TGF1b receptor and activates its non-canonical Erk, c-Jun amino-terminal kinase (JNK) pathway [52,53,54]. JNK’s main role is to promote apoptosis, cross-talking with NF-kb pathway. However, it is also notoriously involved with oncogenic transformation: high levels of JNK activity were found in several cancer lines [55]. By TRAF6 post-transcriptional regulation, miR-146a could also be capable of inhibiting the JNK pathway. Pro-inflammatory cytokines such as IL-8 and IL-1b too are involved in positive regulation of the JNK cascade. ADNV-induced reduction in IL-8 and IL-1b expression coupled with the surge in miR-146a transcription could therefore decrease JNK pathway activation. Furthermore, ADNVs-treated samples induced heavy upregulation of miRNA-30a-5p, a tumor-suppressing miRNA involved in cell cycle progression. Tumor suppression is reached by the regulation of E2F7 proteins in gallbladder and esophageal cancer, and GRP78 in renal carcinoma [56,57,58,59]. miRNAs hsa-miR-652-3p and hsa-miR-331-3p are instead tumor-inducing miRNAs, and they were both less transcribed in ADNV-treated samples (Figure 5C) [60,61,62,63]. 

## 5. Conclusions

An important source of clinical therapeutics is represented by natural active components thanks to their properties associated with multipharmacological activity. This complexity and variability make the definition of their pharmacological profile difficult. Last but not least, there is a lack of published results related to their immunomodulatory effects, inducing limitations in developing new promising entities. To improve the pharmacokinetic profile of these molecules, novel alternative strategies have been developed: the development of nanotechnology-based delivery systems, such as nanoparticles. In this view we must direct attention to the fact that natural compounds such as apples are particularly rich in natural nanovesicles with high clinical potential thanks to their pharmacological activities: the exosomes. In this research we not only were able to isolate and characterize them, but we also performed a well-detailed assessment of their biological activity in vitro in order to support the well-known properties of the apples and their ability to “keep away the doctor”. In light of our results, we can indeed assume that exosomes derived from apples show an ability to talk directly with the immune system, driving it in anti-inflammatory behavior. For this reason, we can speculate that the daily intake of apples allows the consumption of small doses of a natural anti-inflammatory, ensuring the control of inflammatory processes, especially if they are at the initial stage. 

## Figures and Tables

**Table 1:** Primers sequences for RT-PCR.

Gene	FOR	REV
IL-1β	CCTGTCCTGCGTGTTGAAAGA	GGGAACTGGGCAGACTCAAA
IL-8	ACTCCAAACCTTTCCACCCC	TTCTCAGCCCTCTTCAAAAACT
ACTB1 ^1^	GCATCCACGAAACTACCTTCAACTC	CTTGATCTTCATTGTGCTGGGTG

^1^ Housekeeping gene (HKG).

**Table 2:** miRNAs and their regulatory roles.

miRNAs	Function (miRNet)
miR-125a-3p	Inflammation; Apoptosis; Tumor-suppressing miRNAs
miR-30a-5p	Innate immunity; Immune response
miR-146a-5p	Regulation of NF-kb; Innate immunity; Immune response; Cell proliferation; Cell death
miR-331-3p	Regulation of AKT pathway; Glucose metabolism; Toxicity
miR-139-5p	T cell differentiation
